# A Synthetic SOD/Catalase Mimic Compound for the Treatment of ALS

**DOI:** 10.3390/antiox10060827

**Published:** 2021-05-22

**Authors:** Matan Soll, Hagit Goldshtein, Ron Rotkopf, Niva Russek-Blum, Zeev Gross

**Affiliations:** 1 Schulich Faculty of Chemistry, Technion–Israel Institute of Technology, Haifa 32000, Israel; ssolliway@technion.ac.il; 2 The Dead Sea & Arava Science Center, Auspices of Ben Gurion University, Central Arava 86815, Israel; hagitadir@gmail.com; 3 Bioinformatics and Biological Computing Unit, Life Sciences Core Facilities, Weizmann Institute of Science, Rehovot 7610001, Israel; ron.rotkopf@weizmann.ac.il

**Keywords:** catalytic antioxidant, ALS, zebrafish, mSod1 model, corroles, therapeutics

## Abstract

Amyotrophic lateral sclerosis (ALS) is a progressive neurodegenerative disease affecting motor neurons. To date, the etiology of the disease is still unclear, with evidence of reactive oxygen species, mitochondrial dysfunction, iron homeostasis perturbation, protein misfolding and protein aggregation as key players in the pathology of the disease. Twenty percent of familial ALS and two percent of sporadic ALS instances are due to a mutation in Cu/Zn superoxide dismutase (SOD1). Sporadic and familial ALS affects the same neurons with similar pathology; therefore, the underlying hypothesis is that therapies effective in mutant SOD1 models could be translated to sporadic ALS. Corrole metal complexes have lately been identified as strong and potent catalytic antioxidants with beneficial effects in oxidative stress-related diseases such as Parkinson’s disease, Alzheimer’s disease, atherosclerosis, diabetes and its complications. One of the most promising candidates is the iron complex of an amphiphilic corrole, **1-Fe**. In this study we used the SOD1 G93R mutant zebrafish ALS model to assess whether **1-Fe**, as a potent catalytic antioxidant, displays any therapeutic merits in vivo. Our results show that **1-Fe** caused a substantial increase in mutant zebrafish locomotor activity (up to 30%), bringing the locomotive abilities of the mutant treated group close to that of the wild type untreated group (50% more than the mutated untreated group). Furthermore, **1-Fe** did not affect WT larvae locomotor activity, suggesting that **1-Fe** enhances locomotor ability by targeting mechanisms underlying SOD1 ALS specifically. These results may pave the way for future development of **1-Fe** as a viable treatment for ALS.

## 1. Introduction

Amyotrophic lateral sclerosis (ALS) is a fatal neurodegenerative disease affecting motor neurons with an incidence of about 1 in every 100,000 people. Most cases of ALS are sporadic and only 10% of the cases are familial. Both forms of ALS are associated with degeneration of cortical and spinal motor neurons. Although ALS is under intensive research, its etiology remains mainly unknown. Mutations of superoxide dismutase 1 (SOD1, the main enzymatic antioxidant involved in cellular redox homeostasis) are nevertheless known as one of the most common causes of familial ALS [1,2]. This finding allowed the establishment of several common ALS disease models that disclosed deeper understanding of the neuronal cell death mechanisms and the finding of potential therapeutic pathways [2,3,4,5,6]. These investigations have highlighted several major mechanisms accompanying the pathogenesis of ALS, which are not mutually exclusive: oxidative stress; excitotoxicity caused by aberrant glutamate signaling; mitochondrial dysfunction; disruption of the neurofilament network and intracellular trafficking along neurofilaments; aggregation of proteins; and involvement of non-neuronal cells in the vicinity of motor neurons.

The cellular oxidation/reduction (redox) states govern and regulate many aspects of cellular functions maintaining homeostasis [7,8,9,10]. Benign levels of reactive oxygen species/reactive nitrogen species (ROS/RNS) function as signals to promote cell proliferation, regulation and survival. Under normal physiological conditions, cells generate ROS/RNS which include free radical species such as superoxide anion radical (O_2_^•−^), hydroxyl radicals (^•^OH), peroxynitrite (ONOO^−^) and hydrogen peroxide (H_2_O_2_). Peroxynitrite is generated from the combination of superoxide anion radical with the product of nitric oxide synthase, nitric oxide (NO) [11]. Under a myriad of disease conditions, cellular redox homeostasis perturbation can be detected, leading to self-propagating formation of ROS/RNS in a vicious cycle playing pivotal roles in disease pathogenesis, amongst others in ALS [12,13]. Increased levels of inducible nitric oxide synthase (iNOS) and 3-nitrotyrosine (a peroxynitrite oxidation biomarker) have been observed in the motor neurons of ALS patients, corroborating the role of RNS in the pathology [14,15]. Peroxynitrite in the protonated form decomposes to hydroxyl radical and radical nitrogen dioxide (NO_2_), which act as strong oxidizing and nitrating species, reacting with vital amino acids (e.g., tyrosine to 3-nitrotyrosine, which is phosphorylated in a myriad of biological signaling pathways), nucleic acids and fatty acids [16,17]. Many studies have reported increased levels of oxidative damage to proteins, lipids and DNA of postmortem neuronal tissue [18,19,20], as well as in cerebrospinal fluid [21,22,23,24], plasma [25] and urine [26] samples collected from ALS patients. In another recent study, researchers found elevated levels of several oxidative stress biomarkers in ALS patients relative to healthy controls and that edaravone (radicava) alleviates some of these measurements [27].

**Scheme 1:**
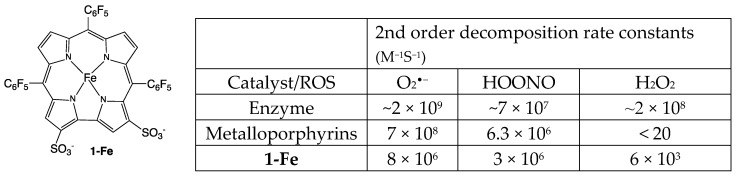
The structure of the corrole-based catalytic antioxidant **1-Fe** and a comparison of its ROS decomposition rates relative to those of the natural enzymes (SOD for O_2_^•−^ and catalase for H_2_O_2_) and porphyrin-based metal (Mn or Fe) complexes [28,29,30,31].

The iron(III) complex of 2,17-bis-sulfonato-5,10,15-tris(pentafluorophenyl)corrole(**1-Fe**) has been disclosed as an excellent catalyst for the decomposition of both ROS and RNS (Scheme 1) [28,29,30,31], including even H_2_O_2_, which is neutralized by **1-Fe** much more efficiently than by any other synthetic catalytic antioxidant. This feature was demonstrated to come into effect for the prevention of cardiovascular diseases, neurodegenerative diseases and diabetes in appropriate animal-based models thereof [29,31,32,33,34,35,36,37,38]. **1-Fe** also displayed phenomenal capabilities in the treatment of optic neuropathies in vivo [39]. In a more recent study, **1-Fe** displayed potent neurorestorative and neuroprotective activity in an in vitro Parkinson’s progressed disease model, restoring basal neuronal and dopaminergic activity even after significant neuronal damage had been inflicted [38]. Furthermore, the disclosed effect was not only due to broad-range antioxidative effects of **1-Fe**, but also due to the neurotrophic properties of **1-Fe,** which activates its neurorestorative effects through neuronal survival signaling and redox modulation by upstream signal transduction activation. In still another in vitro neurodegenerative model, **1-Fe** disclosed high affinity to amyloid-β, the Alzheimer’s-disease-associated neurofibrillary tangles, and prevented both the formation of copper-induced ROS formation and the large aggregated protein plaques associated with the disease [40]. In contrast with dietary antioxidants (e.g., resveratrol, EGCG, curcumin etc.), which act on a 1:1 stoichiometry, a single molecule of **1-Fe** may detoxify numerous ROS/RNS in a catalytic and efficient manner. In effect, **1-Fe** acts as SOD, catalase and theoredoxin enzyme mimetic compound. Therefore, an examination of such a broad-range catalytic antioxidant in an established ALS model may significantly contribute to the debate surrounding the role of redox homeostasis in ALS and the exploration of its potential as a therapeutic agent.

For rapid screening of potential drugs that will slow/inhibit the progression of the disease, a high-throughput system that will faithfully recapitulate the disease phenotype is required. Although mice are still considered the gold standard for drug screening, zebrafish as an animal with complex vertebrate biology offers a powerful model: a combination of easily manipulated, fast-growing and transparent embryos enables rapid in vivo analysis. Zebrafish models for ALS were hence generated and it has repeatedly been shown that they can complement existing mammal models [41,42]. SOD1 G93R mutated transgenic zebrafish (the top ALS-linked gene) were used in this study since it was previously shown that they recapitulate the major phenotypes of ALS, including neuromuscular junction defects, motor neurons loss and muscle pathology [42,43]. Moreover, as evident in humans, the onset and progression of the disease in the zebrafish model is variable, which may reflect a more natural state of the disease, as seen in humans. Several studies show that this model can be used as a chemical and/or genetic screening tool [43,44,45].

In this study, we demonstrate the potential of **1-Fe** as a therapeutic agent for ALS by administration of this potent catalytic antioxidant to an ALS SOD1 G93R zebrafish model. **1-Fe** supplementation induces substantial improvement in the swimming activity of SOD1 mutant zebrafish, in a dose-dependent manner.

## 2. Materials and Methods

### 2.1. Zebrafish

Adult and larval zebrafish (*Danio rerio*) were bred and reared at 28.5 °C under 12 h/12 h light/dark cycle, according to standard protocols [46]. All experiments using Tg(SOD1:SOD1G93R) zebrafish were approved by the Ben Gurion University Committee of Use and Care of Animals and conducted at the ADSSC institute, Israel. Tg(SOD1:SOD1G93R) line was kindly provided by Prof. Christine E. Beattie [42].

### 2.2. Drug Administration Protocol

Wild type (WT) or mutant SOD1 (mSOD1) fish were treated with distinct concentrations of **1-Fe**, including a 0.1% DMSO as background. **1-Fe** was dissolved to a stock solution of 1 mM in ddH_2_O. The compound was diluted in zebrafish raising buffer [46]. Larvae were treated at 3 days post fertilization (dpf) and the solutions were replaced by fresh ones at 5 dpf.

### 2.3. Toxicity Evaluation

Following treatments, fish were observed so as to evaluate possible toxicity. Acute toxicity such as apoptosis/necrosis, specific organ toxicity (liver, kidney, head, eyes, etc.), cardiovascular system abnormalities (heart rate, morphology, hemorrhage and edema) and behavioral toxicity were recorded according to accepted procedures [47].

### 2.4. Motor Performance of SOD1 G93R Larvae

The DanioVision tracking system (Ethovision XT 13.0; Noldus Information Technology, the Netherlands) was used for swimming measurements. Each animal was tested for its x,y position using dynamic subtraction taking 30 frames per second. Larvae were evaluated for locomotor activity at 6 dpf. Individual larvae were placed in 48-well plates, which were put in the DanioVision system with light on for 20 min prior to the beginning of the trial. Larvae were subjected to 10 min dark followed by 10 min light. Larval activity was measured and analyzed during the last 10 min light period, measuring recovery from dark/light transition. Each tested plate contained control animals from the same spawn. Each experiment was repeated using distinct spawns. Experiments were conducted at the same time of day (10:00–14:00) at 24–25 °C. Activity parameters were extracted to Excel and analyzed using Access and R.

### 2.5. Statistics

Total swimming distances (calculated in 1 min time bins) were averaged and compared between treatments using a linear mixed effects model, with treatment as a fixed effect and a random intercept for each plate. A Tukey post hoc test was used to compare all treatments. Additionally, all distances (averaged per fish) were scaled per plate divided by the control mean. These scaled distances were compared between treatments by a one-way ANOVA, followed by a Tukey post hoc test. Statistics were conducted using the R, v.3.6 software.

## 3. Results

**Figure 1:**
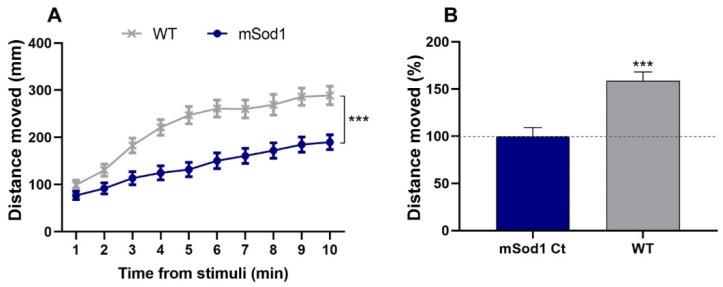
SOD1 G93R mutant locomotor activity. (**A**) The distances that 6 dpf WT and mSOD1 larvae swam following light stimuli per 1 min time bin were measured and averaged (*** *p* < 0.001; linear mixed effect model; *n* = 48/group). (**B**) The change (%) in the distance that the WT larvae swam compared to mSod1 larvae. The distances were averaged per fish for the whole 10 min period following light stimuli, scaled per plate and compared between conditions (*** *p* < 0.001; linear mixed effect model; *n* = 48/group).

The SOD1 G93R mutant zebrafish (referred to herein as mSod1), which integrates the characteristic pathophysiology hallmarks of ALS, was used in a drug-screening platform to evaluate the toxicity and efficacy of the iron corrole **1-Fe**. In this system, the locomotor ability of the larvae was measured following dark/light transition using an automated high-throughput tracking device. The averaged distance that the larvae swam per time bin of 1 min following light stimuli was calculated and this analysis showed that mSod1 larvae swam significantly shorter distances compared to their WT larvae counterparts (*p* < 0.001; Figure 1A,B).

**1-Fe** was first evaluated using screening concentrations ranging between 0.01 and 100 μM. This dose range was shown previously to identify the highest concentrations that can lead to toxicity and death in zebrafish, and is low enough to identify weak active agents [45]. To evaluate **1-Fe** toxicity, we first introduced it to the swimming water of 3 and 5 dpf mSod1 larvae in final concentrations of 100, 10, 5, 1, 0.1 and 0.05 μM. All experiments were conducted with the background of 0.1% DMSO in all samples (including the control sample as a vehicle). Toxicity was evident at 100 and 10 μM **1-Fe** treatments: following the first 10 min of 100 μM **1-Fe** treatment, 100% of both mSod1 and WT larvae were dead. Following the 10 μM **1-Fe** treatment, larvae exhibited decreased heart rate and severe behavioral toxicity with significantly slower and weaker movements in both strains; 40–45% were dead (Table 1).

Drug-induced effects were not observed on gross morphology or mortality in mSod1 larvae treated with 5 μM or lower **1-Fe** concentrations. Still, WT larvae treated with 5 μM **1-Fe** did exhibit a reduction in swimming ability, while 1 μM or lower **1-Fe** concentrations exhibited no obvious drug-induced toxicity, as in mSod1 (Table 1). This suggests that mSod1 **1-Fe**-treated larvae were slightly less sensitive to **1-Fe** toxicity. Future investigation into the underlying mechanisms would disclose the reasons for this phenomenon.

**Figure 2:**
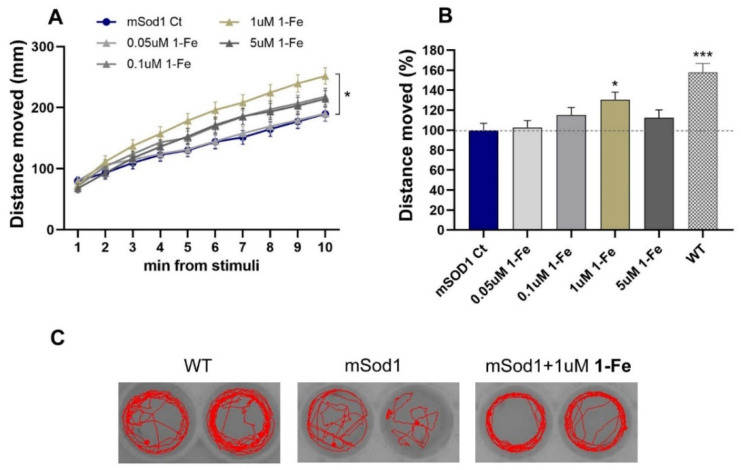
**1-Fe** treatment improved mSOD1 larvae motor performance. (**A**) The distance that mSOD1 larvae swam per time bin of 1 min following light stimuli was averaged for all larvae. mSOD1 larvae were treated with either vehicle (0.1% DMSO; Ct), 0.05, 0.1, 1 or 5 μM **1-Fe** (* *p* < 0.05; linear mixed model, Tukey post hoc test, *n* = 94-6/group). (**B**) The change (%) in the distance the mSOD1-treated larvae swam compared to their vehicle-treated mSOD1 counterparts. The distances were averaged per fish for the whole period following light stimuli, scaled per plate and compared between treatments (* *p* < 0.05; *** *p* < 0.001; one-way ANOVA, Tukey post hoc test; *n* = 94-6/group). (**C**) Characteristic swimming paths of individual larvae for 60 s. Restoration of the swimming pattern was observed following treatment with **1-Fe**.

Further efficacy analyses revealed that treatment with 1 μM **1-Fe** significantly improved locomotion in mSod1 G93R larvae (*p* < 0.05; Figure 2A). Comparison of the effects of the treatments on mSod1 fish motor abilities, averaged for the whole 10 min period, revealed that treatment with 1 µM **1-Fe** induced a significant increase of 30.5% in locomotor activity of mSod1 larvae (*p* < 0.05; Figure 2B,C). SOD1 mutants treated with the lower applied concentration of 0.05 µM and 0.1 µM **1-Fe** or higher concentration of 5 µM **1-Fe** showed a lesser, non-statistically significant increase in their swimming ability (2.7, 15.2 and 12.4%, respectively), forming a bell-shaped dose-response curve (Figure 2B).

These results suggest that the 1 μM **1-Fe** dose is optimal, and that higher doses may reflect compensation between improved swimming ability and a subtle toxic effect.

**Figure 3:**
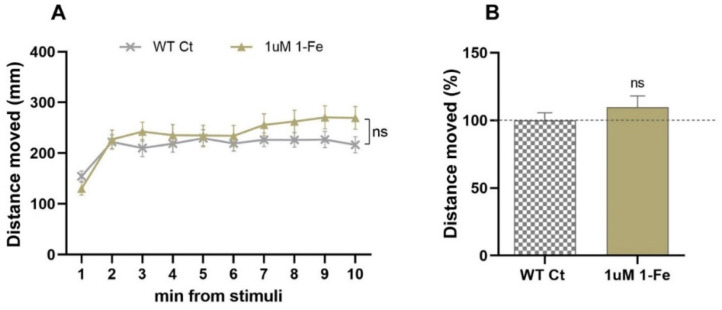
**1-Fe** treatment did not affect motor activity of WT larvae. (**A**) The distance vehicle-treated (0.1% DMSO; Ct) or 1 μM **1-Fe**-treated WT larvae swam, per time bin of 1 min following light stimuli was averaged (ns = non-significant; linear mixed effect model, *n* = 48/group). (**B**) The change (%) in the distance the WT-treated larvae swam compared to their vehicle-treated WT counterparts. The distances swam were averaged per larva for the whole 10 min following light stimuli, scaled per plate and compared between treatments (ns = non-significant; linear mixed effect model, *n* = 48/group).

In order to find out whether the locomotor activity enhancement was specific to the ALS model, WT larvae were subjected to treatment with the optimal 1 μM **1-Fe** dose. The **1-Fe** treatment did not affect WT larvae swimming distance (Figure 3A,B), suggesting that the **1-Fe** treatment enhances locomotor ability in SOD1 ALS specifically, affecting ALS underlying mechanisms.

## 4. Discussion

A plethora of evidence suggests the involvement of ROS/RNS in the pathogeneses of ALS. The involvement of ROS/RNS is one of the core reasons for the self-propagating feature of the neurodegenerative process, as ROS/RNS may form misfolded proteins in the endoplasmic reticulum (ER) and ablate key biological processes that in turn produce more ROS/RNS or vice versa [48,49,50]. Recent evidence of a prion-like spreading in ALS suggests that this self-propagated mechanism can spread further into the nervous system [51].

Iron dysregulation is emerging as a key phenotype in the pathogenesis of ALS [52]. A new meta-analysis by Lang W. and coworkers suggests that ALS patients display no difference in total iron pool relative to control healthy patients [53]. In contrast, there is a staggering difference in the levels of ferritin and transferrin. ALS patients display higher levels of ferritin and lower levels of transferrin, thus substantiating the evidence for dysregulated iron homeostasis in ALS patients. This is corroborated by the increased incorporation of iron into microglia cells deep within the motor cortex of some ALS patients, leading to hypointense signals under MRI imaging [54]. The source of the iron accumulated in microglia cells may be attributed to the major roles of microglia as scavengers of plaques; damaged or unnecessary neurons/synapses; and infectious agents [55]. Though not much is known on the relationship between ROS/RNS and iron dysregulation in ALS patients, other works suggest a direct relationship between oxidative stress and increased levels of unincorporated iron. The pivotal role of Fe-S clusters in the basal activity of the mitochondria in humans and in mitochondrial dysfunction in ROS-related neurodegenerative diseases exacerbates the possible role it co-plays both in iron dysregulation and mitochondrial dysfunction [56]. The release of free iron is also prominent under oxidative stress from hemoglobin and its derivatives in various conditions, suggesting that unregulated redox states induce demetallation of essential iron proteins/enzymes, leading to exacerbation of oxidative stress through Fenton reactions [57]. The elevated levels of ferritin in ALS patients suggest the increase of unincorporated iron (II) levels and act as a marker for enhanced oxidative stress.

The reported increased levels of oxidative stress in ALS may also explain the involvement of astrocytes and its cytotoxicity towards motoneurons [58,59]. The presence of reactive astrocytes and microglia cells in ALS, together with mild infiltration of peripheral immune cells, constitutes one of the major contributors to the formation of ROS/RNS in the central nervous system [60].

**1-Fe** has proven its effectiveness in several conditions where redox dysregulation and inflammation are tightly involved [31]. Amongst others, this compound has shown significant merits in models of Parkinson’s disease, neuropathy, diabetes, atherosclerosis and Alzheimer’s disease [31,32,33,34,35,36,37,38,39,40]. In this research, we have disclosed the substantial efficacy of **1-Fe** in an SOD1 G93R mutated zebrafish model. The broad activity of **1-Fe**, not only decomposing a myriad of ROS/RNS in an effective catalytic manner but also activating neurotrophic pathways [38], may serve as neuroprotective in this in vivo model of ALS. The ability of **1-Fe** to mitigate the levels of ROS/RNS, in effect modulating redox signaling, somewhat allows the cessation of the self-propagating process of oxidative stress that leads to neuroinflammation and cell death. **1-Fe** accumulates mainly in the mitochondria of neurons, with some accumulation in other cellular compartments, allowing the mitigation of ROS formation and cell death via mitochondrial dysfunction and cell death pathways [38]. The effectiveness of **1-Fe** in an in vitro Alzheimer’s disease model has also been disclosed, where β-amyloid aggregation and deleterious pro-oxidant copper binding have been prevented by **1-Fe** administration [40]. β-amyloids accumulate in the anterior horn motor neurons of ALS patients [18]; therefore, **1-Fe** may prevent protein aggregation and may affect the loss of normal biochemical and biological processes (e.g., neurofilament network and intracellular trafficking along neurofilaments). The mounting evidence of oxidative stress as detected by biomarkers from the cerebrospinal fluid, plasma and urine of sporadic ALS patients suggests that the oxidative stress is systemic [52]. Therefore, **1-Fe** could prove itself to be a potential treatment of ALS systemically. In this research, we have disclosed the substantial efficacy of **1-Fe** in an SOD1 G93R mutated zebrafish model. Following our compelling locomotor activity assays, future studies may reveal the exact targets and mechanism of **1-Fe** and its relevance to ALS. Future efforts may lead to the use of **1-Fe** as a therapeutic agent in a clinical setting as a viable treatment for ALS.

## 5. Conclusions

In this study, we have assessed the potential of **1-Fe** to be used as a therapeutic agent for the treatment of ALS. This study suggests that **1-Fe** administration may affect ALS progression and motor neurons’ functional deterioration. Future efforts may lead to clinical adaptation of **1-Fe** as a systemic catalytic antioxidant for the treatment of ALS. 

## Figures and Tables

**Table 1:** Toxicity evaluation of **1-Fe** treatment in WT and mSod1 larvae.

Material	Concentration	Survival % (I treatment; t = 0)		Survival % (I treatment; t = 24 h)		Survival % (II treatment; t = 72 h)		Toxicity	
		WT	mSod1	WT	mSod1	WT	mSod1	WT	mSod1
**1-Fe**	100 μM	100	100	0	0	0	0	death	death
	10 μM	100	100	100	100	40	45	decreased heart rate, severe behavioral toxicity with significantly slower and weaker movements and death	decreased heart rate, severe behavioral toxicity with significantly slower and weaker movements and death
	5 μM	100	100	100	100	100	100	mild behavioral toxicity	no obvious drug induced effects
	1 μM	100	100	100	100	100	100	no obvious drug induced effects	no obvious drug induced effects
	0.05 μM	100	100	100	100	100	100	no obvious drug induced effects	no obvious drug induced effects

## Data Availability

The data presented in this study are openly available in FigShare at https://doi.org/10.6084/m9.figshare.14632725, accessed on 18 May 2021.
